# Reproducibility determination of WHO classification of endometrial hyperplasia/well differentiated adenocarcinoma and comparison with computerized morphometric data in curettage specimens in Iran

**DOI:** 10.1186/1746-1596-4-10

**Published:** 2009-03-25

**Authors:** Narges Izadi-Mood, Maryam Yarmohammadi, Seyed Ali Ahmadi, Guity Irvanloo, Hayedeh Haeri, Ali Pasha Meysamie, Mahmood Khaniki

**Affiliations:** 1 Department of pathology, Mirza Koochak Khan Hospital, Tehran University of Medical sciences, Tehran, Iran; 2 Department of pathology, Sina Hospital, Tehran University of Medical Sciences, Tehran, Iran; 3 Department of pathology, Imam Khomeini Hospital, Tehran University of Medical sciences, Tehran, Iran; 4 Department of Community and Preventive Medicine, Medical school, Tehran University of Medical sciences, Tehran, Iran

## Abstract

**Background:**

Management of endometrial precancerous lesions has been of much debate due to inconsistencies in their classification, natural history and histologic diagnosis. Endometrial hyperplasia constitutes a wide range of histomorphologic features associated with high intra and interobserver diagnostic variability.

Although traditional microscopic diagnosis is by far the most applicable method and the gold standard for histomorphologic diagnosis, digitized image analysis has been used as a powerful adjunct to maximize the histologic data retrieval and to add some detailed objective criteria for correct diagnosis in difficult cases.

**Methods:**

A series of 100 endometrial curettage specimens with diagnosis of endometrial hyperplasia or well differentiated adenocarcinoma were blindly reviewed by 5 pathologists; their intra and interobserver reproducibility determined and further compared to the objective morphometric data i.e. D-score and volume percent of stroma (VPS).

**Results:**

The results were assessed using the weighted kappa statistics. Mean intraobserver kappa value was 0.8690 (99.44% agreement). Mean interobserver kappa values by diagnostic category were: simple hyperplasia without atypia: 0.7441; complex hyperplasia without atypia: 0.3379; atypical hyperplasia: 0.3473, and well-differentiated endometrioid carcinoma: 0.6428; with a kappa value of 0.5372 for all cases combined.

Interobserver agreement was in substantial rate for simple hyperplasia (SH) and well differentiated adenocarcinoma (WDA) but was in fair limit for complex hyperplasia (CH) and atypical hyperplasia (AH). Intraobserver agreement was almost perfect. The specimens were divided in two groups according to the computerized morphometric analysis: Endometrial Hyperplasia (EH) ( D Score ≥ 1 or VPS ≥ 55%) and Endometrial Intraepithelial Neoplasia (EIN) (D-Score < 1 or VPS < 55%). Morphometric findings were closely compatible with routine WHO classification made by one expert pathologist; however; diagnosis of (CH) and (AH) made by other pathologists were not concordant with morphometric data.

**Conclusion:**

It may be necessary to make some revisions in WHO classification for endometrial hyperplasia and precancerous lesions.

## Background

Endometrial hyperplasia which is believed to increase the risk of endometrial carcinoma, is a common disease and comprises a wide spectrum of histological changes from simple aggregation of the normal-looking proliferate glands at one extreme to the changes that are difficult to distinguish from carcinoma at the other end of the spectrum. [1]

The current classification, introduced by Kurman et al 1985, has been accepted by WHO and ISGP. This classification considering two criteria (i.e. glandular complexity and nuclear atypicality) there are four diagnostic categories of endometrial hyperplasia: simple hyperplasia (SH), complex hyperplasia (CH), simple atypical hyperplasia (SAH) and complex atypical hyperplasia (CAH). [2-4]

The wide range of histomorphologic presentation of endometrial hyperplasia is accompanied by high intra and interobserver variability in diagnostic classification. [5]

Previous studies have shown that only 10–20% of endometrial hyperplasias progress to carcinoma when left untreated. [1]

The lack of criteria that could accurately predict the disease outcome may have been an important cause of over and under treatment and need for establishment of a new classification composed of three groups: endometrial hyperplasia (EH), endometrial intraepithelial neoplasm (EIN) and endometrial carcinoma. [5] EIN is defined as a neoplastic focal lesion with cytological features of crowded gland architecture, and a volume percentage less than 55%, with a minimum size of 1 mm and careful exclusion of mimics. [6,7]

This alternative strategy that is intended to recognize the precancerous lesions earlier provides through multivariate analysis, a subset of objectively measured morphometric parameters which may predict the subsequent development or concurrent carcinoma. Several attempts have been made to improve the microscopic tissue diagnosis by the aid of the modern digitized image technology. For example, Kayser et al worked on a method of automatically scanning and analyzing routinely stained glass slides known as virtual microscopy that provides fast and reproducible data about the object-associated (e.g., cells and their nuclei) and non-object-associated (background) tissue components or so-called texture analysis. [8]

Studying on 896 lung cancer slides and using virtual microscopy they produced non-overlapping compartments on each slide that were subsequently subjected to texture analysis. With certain calculations performed at different objective magnifications, they conclude that this system is a fast and reliable procedure for automated pre-screening of lung tumor pathology with diagnostic accuracy of 96–100% that can be made on only 10% of the original image field without increasing error rate. [8]

An additional advantage of digitized image technology is its application in the web-based internet communications also known as telepathology. [8]

Improved image analysis incorporates computer-measure architectural as well as cytological features into a cancer predictive formula (D-Score) which is useful for patient management. [9]

The D-Score has been developed in the early 1980s and its essential features are of architectural (volume percentage stroma and outer surface density of glands) and cytological (standard deviation of the shortest nuclear axis) nature. [5]

Retrospective studies in the USA, the Netherlands and Norway confirmed the prognostic value of the D-Score greatly exceeding the WHO 94 criteria. [5]

D-Score have higher sensitivity (100%), specifity (82%), positive predictive value (PPV 38%) and negative predictive value (NPV 100%) compared to WHO 94 with sensitivity (91%), specifity (58%), PPV (16%) and NPV (99%). [5]

Molecular genetic studies have shown that endometrial lesions with a D-score less than 1 are often monoclonal physical progenitors of subsequent endometrial adenocarcinoma whereas those with a D-score higher than 1 are virtually polycolonal. [6,9]

The PTEN tumor suppressor gene is the most frequently inactivated gene in the premalignant and malignant phases of endometrioid endometrial cancer. [10,11]

Baak et al revealed EIN lesions that have lost PTEN tumor suppressor function confer a greater cancer risk compared with EIN lesions with an intact PTEN gene. [6]

## Materials and methods

A retrospective review of the archives of the Department of Pathology of Mirza Kolchak Khan Hospital, for the period of 2001 through 2005 identified 100 patients who had D&C specimens diagnosed as endometrial hyperplasia and well differentiated adenocarcinoma.

The material was fixed in buffered formaldehyde, embedded in paraffin wax and standard hematoxylin eosin (H&E) stained histological sections were made.

Five pathologists with varying experiences in the field of gynecologic pathology who worked at hospitals in Tehran University of medical sciences contributed to this study.

**Figure 1:**
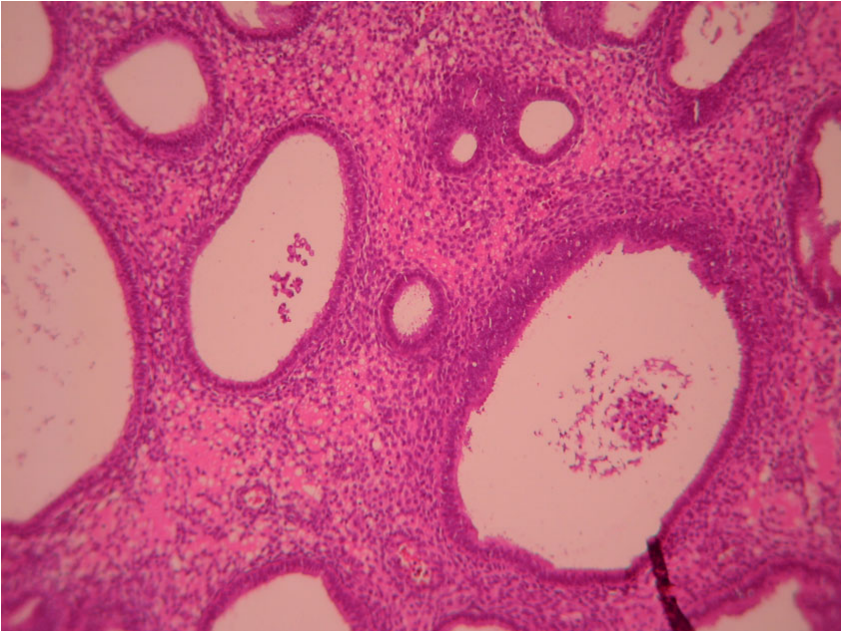
**Simple hyperplasia**. Glands are in various size with occasional dilated, cystic glands separated by abundant stroma. (Low power).

**Figure 2:**
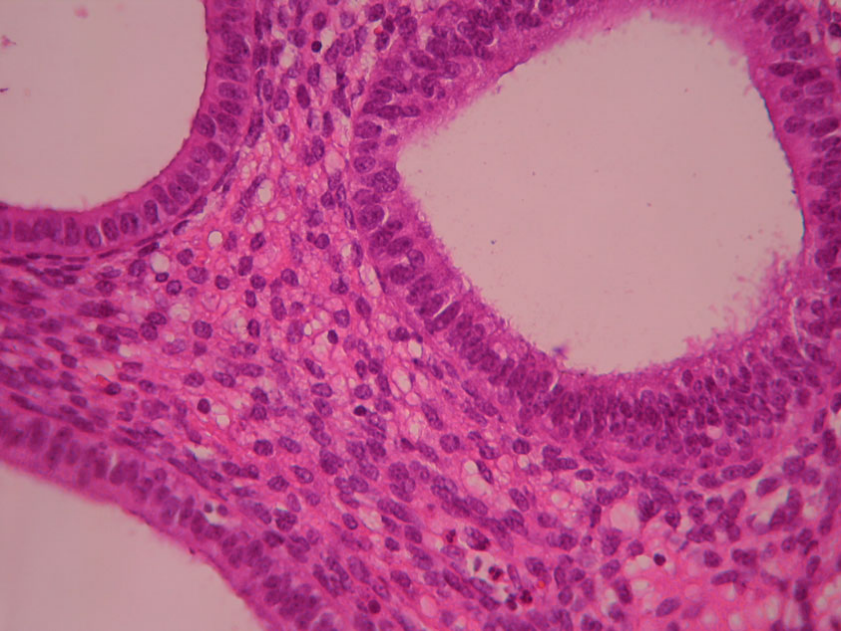
**Simple hyperplasia**. Glands are lined by pseudostratified uniform and oval nuclei. (High power).

**Figure 3:**
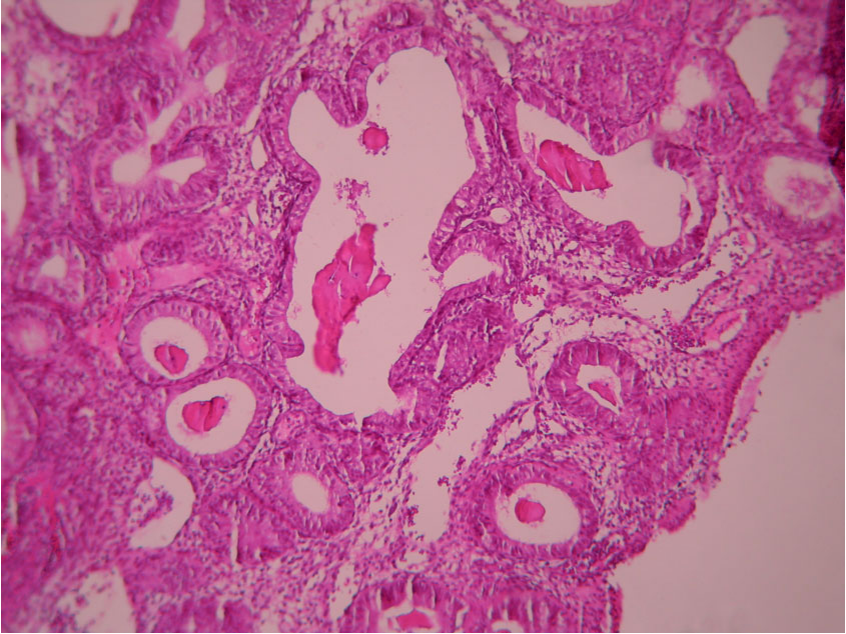
**Complex hyperplasia**. The glands are closely back to back with scant intervening stroma and highly irregular in size and shape. (Low power).

**Figure 4:**
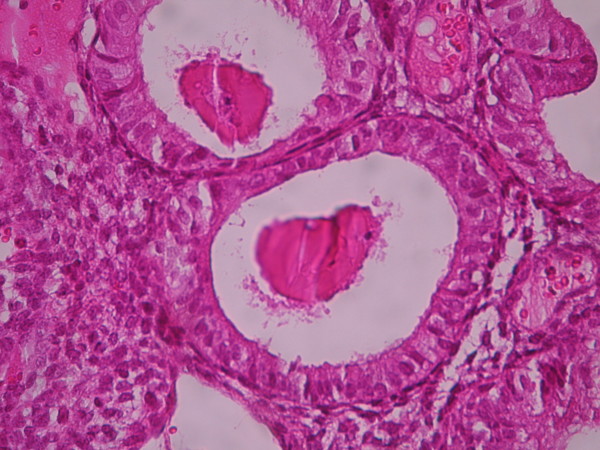
**Complex hyperplasia**. The glands are separated by scant endometrial stroma from each other. The nuclei are uniform and oval. (High power).

**Figure 5:**
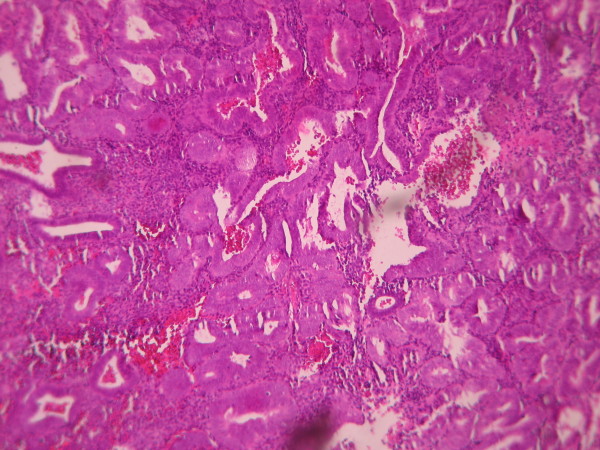
**Atypical hyperplasia**. The glands are closely back to back with scant intervening stroma and highly irregular in size and shape. (low power).

**Figure 6:**
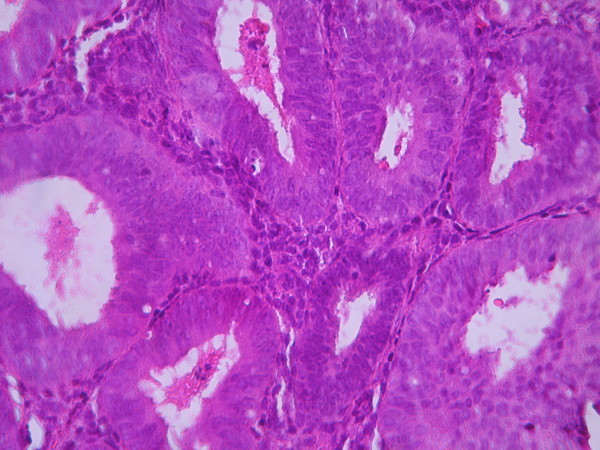
**Atypical hyperplasia**. Back to back glands show stratified round nuclei with nucleoli. (High power).

**Figure 7:**
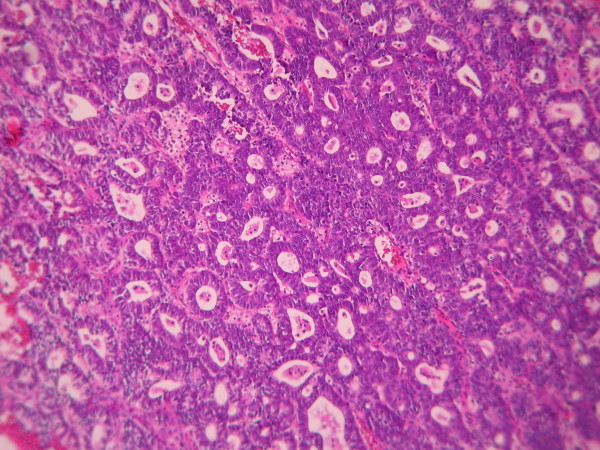
**Endometrial adenocarcinoma**. Closely back to back glands without intervening endometrial stroma and with granulation tissue like stroma and cribriform pattern. (Low power).

**Figure 8:**
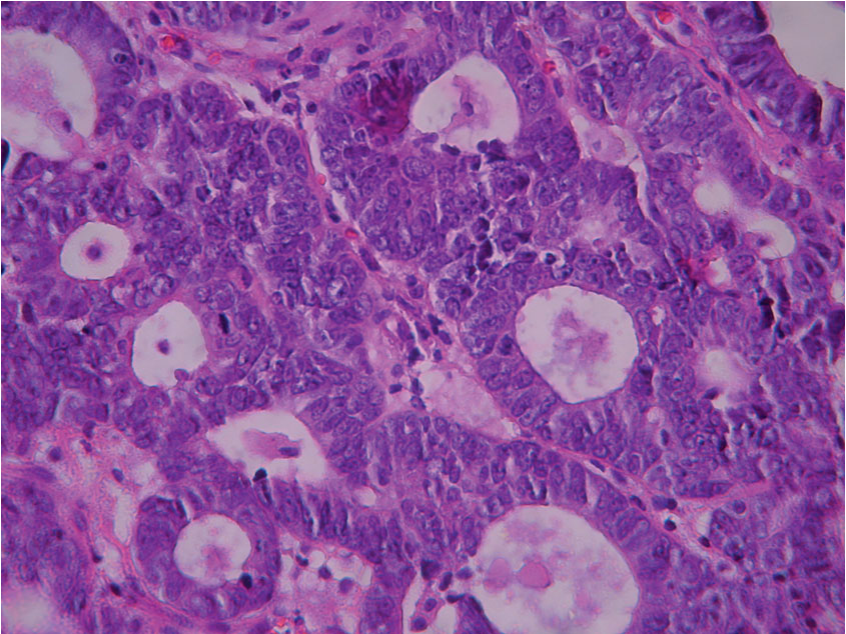
**Endometrial adenocarcinoma**. Confluent growth pattern of glands with no intervening endometrial stroma or loose granulation tissue like stroma. (High power).

**Figure 9:**
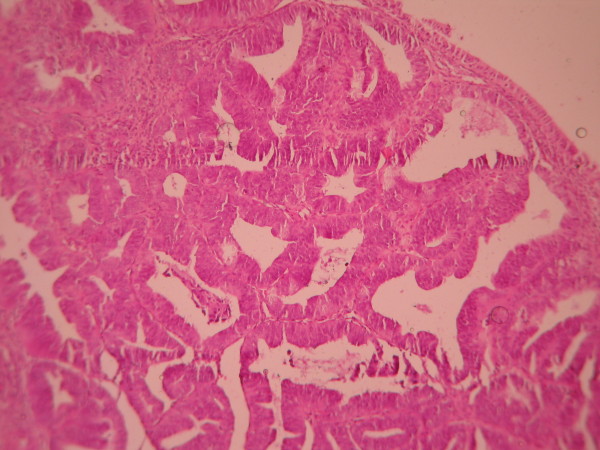
**Endometrial adenocarcinoma**. Confluent growth pattern of glands with no intervening endometrial stroma. (Low power).

**Figure 10:**
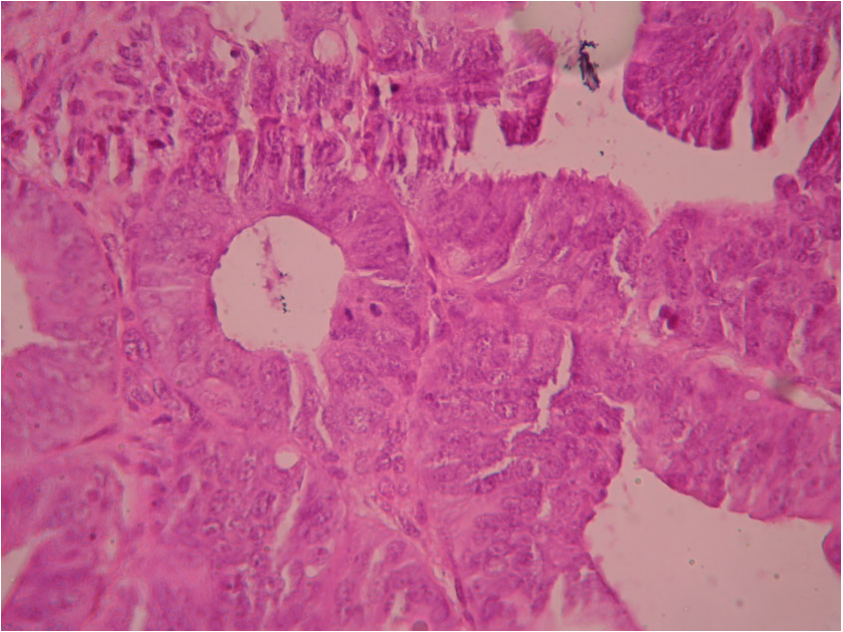
**Endometrial adenocarcinoma**. Confluent growth pattern of glands with no intervening endometrial stroma and severe nuclear atypia. (High power).

**Figure 11:**
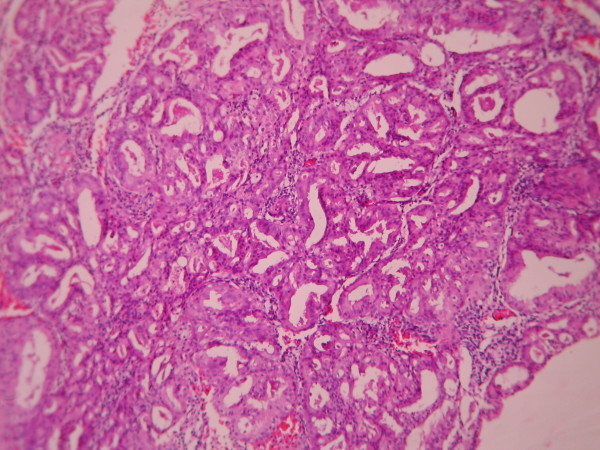
**Endometrial adenocarcinoma**. Cribriform pattern of glands. (Low power).

**Figure 12:**
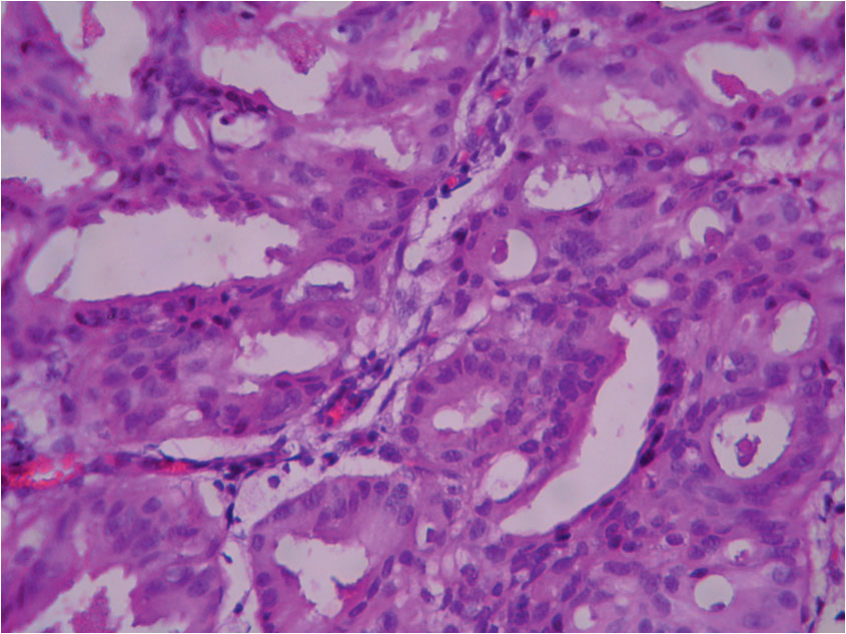
**Endometrial adenocarcinoma**. The glands show cribriform bridging. (High power)

**Figure 13:**
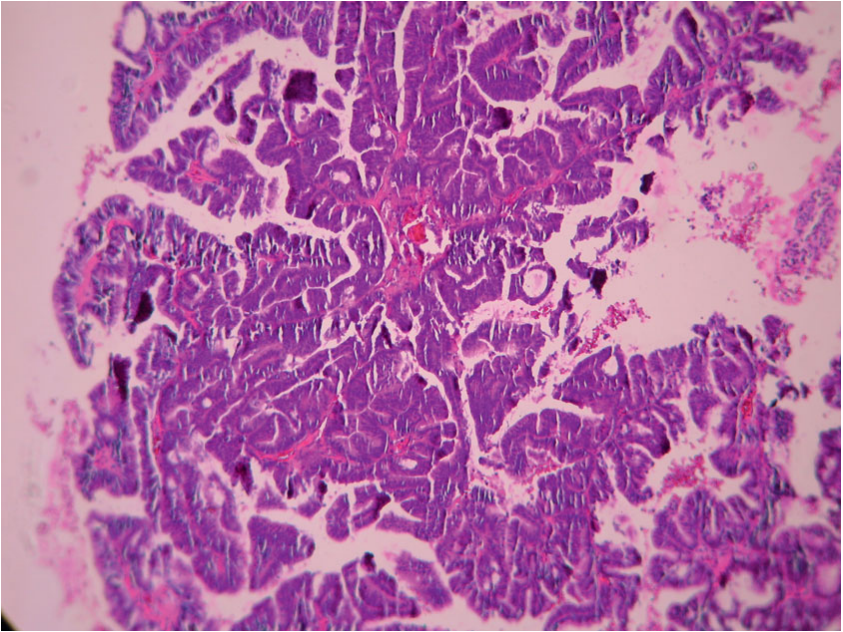
**Endometrial adenocarcinoma.** Extensive papillary pattern. (Low power)

**Figure 14:**
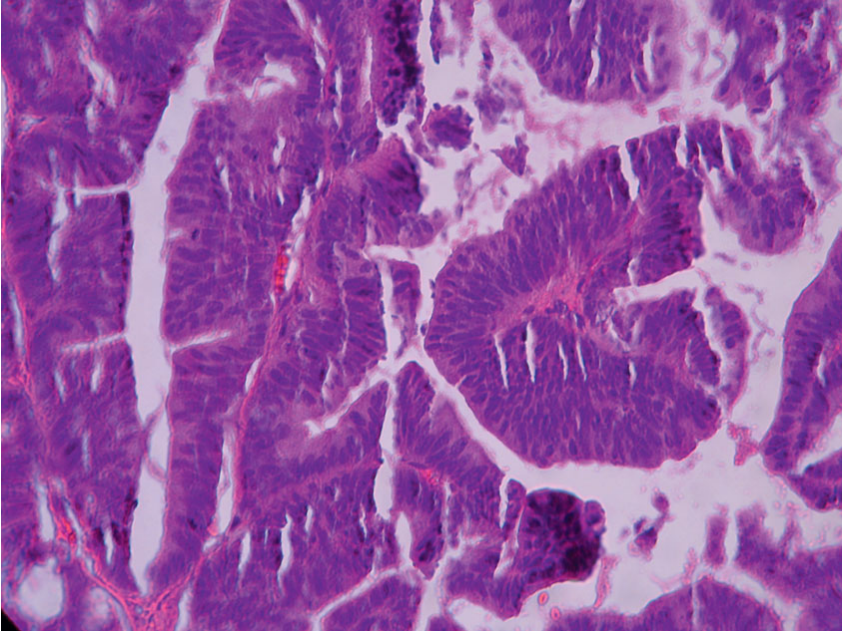
**Endometrial adenocarcinoma**. Papillary growth pattern of glands with stratified nuclei and mild nuclear atypia (High power)

The cases were selected to represent four diagnostic categories including simple hyperplasia(SH) which shows glands are irregular in size and shape with occasional dilated, cystic glands lined by pseudostratified uniform and oval nuclei showing orientation toward the basement membrane and separated by abundant stroma (fig 1 and 2), complex hyperplasia(CH) composed of closely spaced glands, highly irregular in size and shape with pseudostratified uniform and oval nuclei (fig 3 and 4), atypical hyperplasia(AH), a complex hyperplasia which cells show atypia including irregular, stratified, rounded nuclei with nucleoli (fig 5 and 6) and well differentiated adenocarcinoma(WDA), when there are confluent glandular pattern, an extensive papillary pattern, cribriform bridging, desmoplastic or granulation tissue like stroma, and highly atypical cells (fig 7 and 8, 9 and 10, 11 and 12, 13 and 14).

Twenty-five cases from each category were included and one representative H&E slide was selected of each case. To assess interobserver variability slides were randomly labeled from 1 to 100, evaluated by 5 pathologists and presumptive diagnoses were recorded in a checklist. For intraobserver evaluation one expert gynecopathologist examined all of the slides twice within a period of two months.

The checklists included four diagnostic categories (SH, CH, AH and WDA). After data collection the checklists were coded and statistically analyzed using the STATA-8 statistical software and weighted kappa test. Data analysis evaluated interobserver and intraobserver agreement using the (Kappa) statistic, a measure of agreement between observers that attempts to correct for chance agreement. Within the positive values of kappa, given interpretations used in this study were scaled as: 0.00–0.20 = slight, 0.21–0.40 = fair 0.41–0.60 = moderate, 0.61–0.80 = substantial and 0.80–1.00 = almost perfect [12]

The correlation coefficient between morphologic data and results of morphometric

analysis is about 80%, [13] so 55 out of 100 H&E slides were selected randomly which yielded appropriate material for morphometric analysis.

Computerized morphometric analysis of delineated regions on H&E stained sections was performed using the Leica IM 500^® ^(V.4.R117) software incorporated into the digitized light-microscope.

For each sample the D-Score was calculated, using three features include 1) volume percentage of stroma (VPS), which assesses the percentage of endometrial tissue composed of stroma (i.e., the inverse of glandular percentage, a measure of crowding) 2) standard deviation of shortest nuclear axis (SDSNA), which reflects nuclear pleomorphism and 3) gland outer surface density (out SD), which is a measurement of basement membrane length about the endometrial glands (measurement of gland complexity) and the following formula:

D-score = 0.6229 + (0.0439 × VPS) – [3.9934 × Ln (SDSNA)] – (0.1592 × outSD)

[3-5,14] Measurement of these features performed on 9–11 images taken from the most representative hyperplastic areas in H & E stained sections with a minimum size of 1 mm and careful exclusion of mimics and non hyperplastic areas [7,14]. Values of D-Score ≥ 1 or VPS ≥ 55% were defined as one group and D-Score<1 or VPS<55% defined as the other.

In brief, VPS was measured on histological images (40 × objective magnification (field diameter 340 μm) with a 88 point grid or graticule (weibel grid with 2-point length 28.3 μm), and the tissue underlying each point was scored visually from the ocular lens of microscope as stroma, epithelium or gland lumen. Results from a total of 400–600 points were tallied and the VPS was calculated as the number of stromal points divided by the total points counted. (Range of 14–75%)

Intersections of gland outer surfaces with calibrated horizontal lines of the weibel grid were tallied and the outer surface density was calculated by underlying formula:



Nuclear morphometry was preformed on at least 150 randomly selected nuclei and the shortest nuclear axis was calculated by sending results to Microsoft Excel^® ^program followed by nuclear mean and SD determination. Measurement was terminated when the coefficient of variation went below 5% (range of 0.68–1.52)

## Results

For interobserver diagnostic agreement, using the diagnosis given by each pathologist on each diagnostic round, kappa results show significant differences in diagnostic groups, with highest agreement in SH and WDA groups and lowest agreement in CH and AH groups. (Table 1)

**Table 1:** Diagnostic interobserver agreement

**Diagnosis**	**Kappa**	**Agreement**	**Probe >Z**
SH	0.7741	Substantial	**<0.0001**
CH	0.3379	Fair	**<0.0001**
AH	0.3473	Fair	**<0.0001**
WDA	0.6428	Substantial	**<0.0001**
**Combined**	0.5372	Moderate	**<0.0001**

SH: Simple Hyperplasia, CH: Complex Hyperplasia, AH: Atypical Hyperplasia, WDA: Well Differentiated Adenocarcinoma

Table 2, lists intraobserver agreement of the expert gynecopathologist on two separate diagnostic rounds. Kappa results show significant agreement in all diagnostic groups.

**Table 2:** diagnostic intraobserver agreement

**Diagnostic agreement**	**Explanatory agreement**	**Kappa**	**Probe>Z**
99.44%	57.60	0.8690	**0.0000**

After pathologist agreement assessment, mean difference of computerized morphometric data (VPS and D-Score) with pathologist diagnosis subgroup, statistically analyzed with post HOC test and shuffle exam for three pathologists.

According to diagnosis in 01t1 (Observer 1 Time 1), O2t1 (Observer 2 Time 1) & O3t1 (Observer 3 Time 1)

Compared assessment of different diagnostic groups with D-Score results show high concordance and ability in O1t1 (Observer 1 Time 1) for classification and differentiation of endometrial hyperplasia subgroups but overlapping results in differentiation of CH with AH and AH with WDA groups in O2t1 (Observer 2 Time 1) and CH with AH in O3t1 (Observer 3 Time 1).

Case by case comparison of computerized VPS (cut-off range 55%) and D-Score (cut-off range 1) with pathologist diagnosis in four diagnostic variables analyzed with kruss-kall Wallis test are shown in tables 3 and 4.

**Table 3:** Agreement table of diagnostic groups' basis on VPS

	**VPSCAT(O1T1)**		**VPSCAT(O2T1)**		**VPSCAT(O3T1)**	
	≥ 55%	<55%	≥ 55%	<55%	≥ 55%	<55%
**SH**	14 (93.3%)	1 (6.7%)	21(95.5%)	1 (4.5%)	22 (91.7%)	2 (8.3%)
**CH**	10 (62.5%)	6 (37.5%)	2 (18.2%)	9 (81.8%)	1 (7.7%)	12 (92.3%)
**AH**	2 (13.3%)	13 (89.7%)	2 (15.4%)	11 (84.6%)	2 (14.3%)	12 (85.7%)
**WDA**	0 (0%)	9 (100%)	*0* (0%)	*8* (100%)	0 (0%)	3 (100%)
**Total**	**26** **(47.3%)**	**29** **(52.7%)**	**25** **(46.3%)**	**29** **(53.7%)**	**25** **(46.3%)**	**29** **(53.7%)**

SH: Simple Hyperplasia, CH: Complex Hyperplasia, AH: Atypical Hyperplasia, WDA: Well Differentiated Adenocarcinoma.

**Table 4:** Agreement table of diagnostic groups basis on D-Score

	**D-Score(O1T1)**		**D-Score(O2T1)**		**D-Score(O3T1)**	
	≥ 1	<1	≥ 1	<1	≥ 1	<1
**SH**	15 (100%)	0 (0%)	22 (100%)	0 (0%)	24 (100%)	0 (0%)
**CH**	11 (68.8%)	5 (31.2%)	4 (36.4%)	7 (63.6%)	2 (15.4%)	11 (84.6%)
**AH**	2 (13.3%)	13 (86.7%)	1 (7.7%)	12 (92.3%)	1 (7.1%)	13 (92.9%)
**WDA**	0 (0%)	9 (100%)	0 (0%)	8 (100%)	0 (0%)	3 (100%)
**Total**	**28** **(50.9%)**	**27** **(50%)**	**27** **(50%)**	**27** **(50%)**	**27** **(50%)**	**27** **(50%)**

SH: Simple Hyperplasia, CH: Complex Hyperplasia, AH: Atypical Hyperplasia, WDA: Well Differentiated Adenocarcinoma.

## Discussion

Endometrial carcinoma is the most common female genital tract malignancy in developed countries. [15] Endometrioid and papillary serous carcinomas have been recognized as two major clinicopathologic subtypes of this cancer. [15]

Endometrioid subtype may arise in background of endometrial hyperplasia at a younger age while the high grade in an older age group. [15]

The WHO 94 endometrial hyperplasia classification system will continue to play an active role in the daily practice of many pathologists but is plagued by poor diagnostic reproducibility and the lack of a solid statistical foundation on therapeutic context.

It is important to characterize high or low risk groups before initiation of therapy, because about 1–28% of hyperplasias progress to carcinoma, depending on the degree of severity. [14]

Considering the combined interobserver agreement level of "moderate" attained in this study and the previously reported results as "fair" by Skov (1997); "substantial" by Kendall (1998) and "moderate" by Bergeron(1999) it seems that WHO 94 classification system needs essential improvements by an entirely new approach rather than minor revisions. [16-18]EIN classification system (EH-EIN-CA) is the best documented alternative based on extensive morphological, genetic molecular and clinical outcome data.

This new molecular genetic-based and morphometric-based classification differs from the WHO 94, which is based entirely on histological findings. [14]

Diagnosis of EIN is possible with assessment of D-Score and VPS morphometrical parameters i.e. lesions with D-Score<1 or VPS<55% are classified as EIN. It should be emphasized that morphometric studies of endometrial hyperplasia have identified a unique multivariate prognostic combination of quantitative architectural and nuclear features that corresponds well with both cancer risk and biologic lesion properties [6,9] but our focus in this study was to assess diagnostic reproducibility and comparison of results with D-Score and VPS rather than to correlate the diagnosis with outcome. Therefore THERE WAS NO GOLD STANDARD. With grouping of different diagnostic subgroups according to D-Score results, interpretative patterns of individual pathologists fell into two distinctive classes:

One with high concordance and 95% rate of confidence interval in all subgroups but those two others with overlapping results, especially in diagnoses of CH with AH that show lowest rate of reproducibility in all studies.

Compared VPS and D-Score results rendered highly concordant replicate results.

Case by case comparison of VPS (cut-off range 55%) and computerized D-Score (cut-off range 1) with pathologist diagnosis is shown in tables 3 and 4. As the histological diagnosis goes from benign (SH) to malignant (WDA) the VPS decreases to <55% and D-Score becomes <1; however; there is a major difference between 3 pathologists in CH category. In other words, the second and the third pathologists (O2 and O3) have probably "under diagnosed" a premalignant or even malignant lesion as CH. This may result in substantially divergent guidance to the gynecologist and incorrect management such as medical therapy instead of hysterectomy.

In conclusion, diagnosis of endometrial hyperplasia and carcinoma with WHO-defined nomenclature may be problematic, mainly due to stylistic differences between individuals and inherent poor reproducibility of the broad range of diagnoses from benign to malignant.

Limitation of borderline or precancerous lesions into one category (EIN) recognized by objective morphometry will probably simplify the diagnosis and improve the patient's management.

Measurement of VPS – by far the most predictive component of the D-score – can be accomplished simply by applying an inexpensive ocular grid into an ordinary microscope eyepiece and counting the specified points on glandular and stromal components.

## Competing interests

The authors declare that they have no competing interests.

## Authors' contributions

NIM is study designer, contributed in histological diagnosis and writing of manuscript, performed morphometrical analysis. MY Writing of the manuscript and performing morphometrical analysis. SAA Histological diagnosis, writing, revising and editing of manuscript. GI and HH Histological diagnosis and writing of the manuscript. APM Data analysis, interpretation of statistical data and writing of the manuscript. MK histological diagnosis. All authors read and approved the final manuscript.
